# Considerations and recommendations for assessment of plasma protein binding and drug–drug interactions for siRNA therapeutics

**DOI:** 10.1093/nar/gkac456

**Published:** 2022-06-10

**Authors:** Sara C Humphreys, John A Davis, Sajida Iqbal, Amin Kamel, Kenneth Kulmatycki, Yanbin Lao, Xiumin Liu, John Rodgers, Jan Snoeys, Adam Vigil, Yan Weng, Christopher M Wiethoff, Matthias B Wittwer

**Affiliations:** PKDM Department, Amgen Inc., South San Francisco, CA 94080, USA; PKS Department, Novartis, Cambridge, MA 02139, USA; PKDM Department, Sanofi, Waltham, MA 02451, USA; Global DMPK, Takeda, San Diego, CA 92121, USA; PKS Department, Novartis, Cambridge, MA 02139, USA; DMPK, Pharmaceutical Candidate Optimization, Bristol-Myers Squibb, Princeton, NJ 08648, USA; Early Development, Alnylam Pharmaceuticals, Inc., Cambridge, MA 02142, USA; PKDM Department, Amgen Inc., South San Francisco, CA 94080, USA; DMPK Department, Janssen R&D, Beerse 2340, Belgium; DMPK, Boehringer Ingelheim Pharmaceuticals Inc., Ridgefield, CT 06877, USA; Early Clinical Development Clinical Pharmacology Department, Pfizer, Cambridge, MA 02139, USA; Drug Disposition, Eli Lilly and Company, Indianapolis, IN 46285, USA; Pharmaceutical Sciences, Roche, Basel CH-4070, Switzerland

## Abstract

At the time of writing, although siRNA therapeutics are approved for human use, no official regulatory guidance specific to this modality is available. In the absence of guidance, preclinical development for siRNA followed a hybrid of the small molecule and biologics guidance documents. However, siRNA differs significantly from small molecules and protein-based biologics in its physicochemical, absorption, distribution, metabolism and excretion properties, and its mechanism of action. Consequently, certain reports typically included in filing packages for small molecule or biologics may benefit from adaption, or even omission, from an siRNA filing. In this white paper, members of the ‘siRNA working group’ in the IQ Consortium compile a list of reports included in approved siRNA filing packages and discuss the relevance of two *in vitro* reports—the plasma protein binding evaluation and the drug–drug interaction risk assessment—to support siRNA regulatory filings. Publicly available siRNA approval packages and the literature were systematically reviewed to examine the role of siRNA plasma protein binding and drug–drug interactions in understanding pharmacokinetic/pharmacodynamic relationships, safety and translation. The findings are summarized into two decision trees to help guide industry decide when *in vitro* siRNA plasma protein binding and drug–drug interaction studies are warranted.

## INTRODUCTION

### Scope

This perspective was prepared by industry members of the Translational ADME Leadership Group siRNA Working Group of the International Consortium for Innovation and Quality in Pharmaceutical Development (IQ; https://iqconsortium.org/). IQ is a not-for-profit organization of pharmaceutical and biotechnology companies with a mission of advancing science and technology to augment the capability of member companies to develop transformational solutions that benefit patients, regulators and the broader research and development community. The purpose of the work is to review published regulatory approval documents and literature to evaluate the relevance, and provide industry recommendations and decision trees regarding inclusion of*in vitro* PPB evaluation and DDI assessments in regulatory packages for siRNA-containing therapeutic candidates.

As of May 2021, there are four approved siRNA therapies: patisiran, givosiran, lumasiran and inclisiran (patisiran European Medicines Agency (EMA) Assessment Report (EMA/554262/2018; https://www.ema.europa.eu/en/documents/assessment-report/onpattro-epar-public-assessment-report_.pdf, patisiran Food and Drug Administration (FDA) Multi-discipline Review (NDA 210922; https://www.accessdata.fda.gov/drugsatfda_docs/nda/2018/210922Orig1s000MultiR.pdf, givosiran FDA Multi-discipline Review (NDA 212194;

https://www.accessdata.fda.gov/drugsatfda_docs/nda/2019/212194Orig1s000MultidisciplineR.pdf), givosiran EMA Assessment Report (EMA/CHMP/70703/2020; https://www.ema.europa.eu/en/documents/assessment-report/givlaari-epar-public-assessment-report_en.pdf, lumasiran FDA Integrated Review (NDA 21410; https://www.accessdata.fda.gov/drugsatfda_docs/nda/2020/214103Orig1s000IntegratedR.pdf, lumasiran EMA Assessment Report (EMA/568312/2020; https://www.ema.europa.eu/documents/assessment-report/oxlumo-epar-public-assessment-report_en.pdf, inclisiran EMA Assessment Report (EMA/696912/2020; https://www.ema.europa.eu/documents/assessment-report/leqvio-epar-public-assessment-report_en.pdf).

Patisiran is formulated as a lipid nanoparticle (LNP), and the other three are *N*-acetyl galactosamine (GalNAc) conjugates. Consequently, the data and discussion in this manuscript are heavily weighted towards these platforms. However, recognizing that the siRNA field is rapidly evolving, we also consider other siRNA-containing platforms that lead to systemic exposure, including but not limited to, siRNA–peptide conjugates, siRNA–antibody conjugates, siRNA–lipid conjugates, and siRNA formulated in novel excipients.

While we acknowledge that siRNA share some similarities with other oligonucleotide therapeutics (ONTs) such as antisense oligonucleotides (ASOs), different ONT modalities have diverse physicochemical properties, mechanisms of action, and absorption, distribution, metabolism, and excretion (ADME) properties. Consequently, non-siRNA ONTs are considered outside the scope of this document. Data comparisons with other ONT modalities are only included where they are deemed relevant to siRNA.

### siRNA mechanism of action

**Figure 1.**
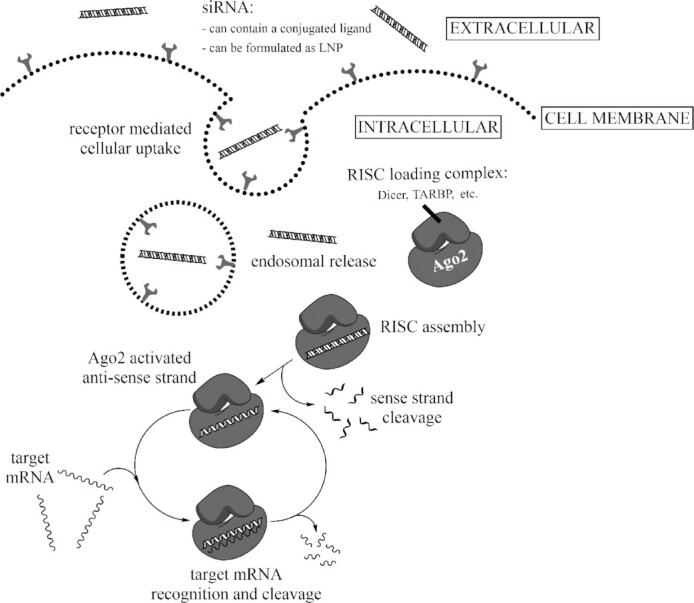
Mechanism of short-interfering (siRNA) uptake and pharmacologic action. Ligand-conjugated siRNA or lipid nanoparticle (LNP)-formulated siRNA is taken up into cells via endosomal pathway. When introduced into cells, siRNA reaches cytoplasm through endosomal release, and then loads into RNA-induced silencing complex (RISC). The antisense strand of siRNA is activated via selective removal of the sense strand by Argonaute 2 (Ago2) and directs recognition of its complementary target mRNA. Ago2 in this enzyme complex cleaves the target mRNA sequence and subsequently suppresses the target protein encoded by it via a catalytic mechanism.

The use of RNA interference as a mechanism for gene silencing has evolved over the last 20 years from a novel research tool to a promising new class of therapeutics for the treatment of a wide array of human diseases. RNA interference describes the process where gene expression is regulated through inhibition of mRNA with small non-coding molecules of RNA. siRNA is a 19–25-mer double-stranded RNA molecule consisting of a pharmacologically inactive sense strand and a pharmacologically active antisense strand. The antisense strand is activated via selective removal of the sense strand by Argonaute 2 (Ago2), an RNA-induced silencing complex (RISC) endonuclease. Antisense-associated Ago2 causes RNA silencing by catalyzing the destruction of and/or selectively inhibiting translation of complementary RNA transcript (Figure 1) (1–3).

### siRNA properties compared to small molecules and protein therapeutics

siRNA is a unique class of therapeutics distinct from small molecule and protein-based therapeutics in both its mechanism of action and physicochemical properties (Table 1). Although it is active intracellularly, cellular uptake of unmodified double-stranded siRNA is limited due to its high molecular weight (∼10–15 kDa) and hydrophilic nature. To enhance uptake, siRNA is often complexed within LNPs or conjugated to a cell-specific targeting ligand, such as GalNAc, which binds to the asialoglycoprotein receptor (ASGPR) predominantly expressed on the cell surface of hepatocytes (4,5).

**Table 1.** Comparison of small molecule, siRNA and mAb therapeutic properties

Property	Small molecule	siRNA	mAb
**Molecular weight**	∼0.5 kDa	∼10–15 kDa	∼150 kDa
**Manufacturing**	chemical synthesis	chemical synthesis	cell line production
**API heterogeneity**	low	medium to high	high
**Physicochemical properties**	typically hydrophobic; neutral, basic, acidic subgroups	hydrophilic; negatively charged at physiological pH	hydrophilic; positive, negative, and neutral sites at physiological pH
**Molecular target**	protein	mRNA	protein
**Selectivity**	lower than the others	high	high
**Site of action**	intracellular and extracellular	intracellular	extracellular (ADCs can be intracellular)
**Cellular uptake**	passive and/or active	formulation-facilitated or receptor-mediated active	via endosomal pathway and recycled or degraded
**Route of administration**	oral or parenteral	parenteral	parenteral
**Dosing frequency**	daily	every three weeks to > monthly	weekly to monthly
**Absorption**	orally bioavailable, fast absorption	not orally bioavailable, s.c. absorption rate fast	not orally bioavailable, s.c. absorption rate slow
**Distribution**	extensive tissue distribution	select tissue distribution	limited to extracellular fluid
**Metabolism**	phase I and II drug metabolizing enzymes mediated	nuclease mediated	protease mediated
**Renal or bile excretion**	frequently	low	no
**Immunogenicity risk**	no	low; minimal impact on PK/PD or safety	yes; and likely to impact PK/PD or safety
**PK/PD**	usually direct correlation between blood PK and PD profile	indirect correlation between blood PK and PD profile; direct correlation between tissue PK and PD profile	direct correlation between blood PK and PD profile
**Onset of action**	fast	delayed	fast
**PD duration**	relatively short	long; could support up to yearly dosing interval	relatively long; weekly to monthly
**DDI risk**	high	low	low
**PPB**	variable; high impact on PK/PD	variable; low impact on PK/PD	low
**Off-target toxicity**	moderate probability	low probability by design	low probability
**Volume of distribution**	variable	extensive distribution to liver	small
**Clearance**	high	rapid plasma clearance, slow tissue clearance	low
**Serum half-life**	short	short	long

Abbreviations: DDI, drug–drug interactions; PD, pharmacodynamics; PK, pharmacokinetics; PPB, plasma protein binding; s.c., subcutaneous; ADC, antibody-drug conjugate.

### ADME and pharmacokinetics/pharmacodynamics of siRNA

All four siRNA therapeutics approved as of May 2021 are administered parenterally and target the liver. Targeted delivery strategies such as GalNAc-conjugation and LNP encapsulation promote rapid tissue distribution and accumulation, resulting in a relatively short plasma elimination half-life of typically minutes to several hours with a much longer tissue half-life of typically days to months (6–9). The major route of clearance from circulation is through tissue uptake, with renal clearance representing a minor clearance pathway. Once in the tissue, the major elimination route has been reported to be nuclease-mediated metabolism (10). Nucleases responsible for metabolism of siRNA are expressed ubiquitously in tissues as well as in systemic circulation. *In vitro* studies suggest metabolism can occur in plasma, serum, liver homogenate, liver microsomes, S9 fractions, and lysosomes, and *in vivo* metabolite formation has been described in mice, rats, monkeys, and humans (11–18). The onset of action of siRNA is typically delayed relative to the time of administration. Since plasma concentrations are transient, the long-lasting duration of pharmacological effect is reflective of target tissue concentration (9). This unique pharmacokinetic/pharmacodynamic (PK/PD) characteristic enables monthly, quarterly, and possibly yearly administration of siRNA to achieve therapeutic efficacy, a dosing frequency not typically achievable for small molecule drugs (10).

### siRNA regulatory and safety considerations as of May 2021

There is no official regulatory and safety guidance specifically for siRNA or ONTs. While nonclinical safety studies for siRNA mainly follow the small molecule guidance, ICH M3(R2), preclinical development follows a hybrid of ICH M3(R2) and the biologics guidance, ICH S6(R1). Although siRNA have been associated with infusion-related and injection-site reactions, they are generally well tolerated. Safety concerns specific to LNP formulated siRNA including cytokine release, renal and hepatic toxicities, are shared with other LNP-formulated ONTs such as ASOs and mRNA (19–21). Recommendations for safety assessment and drug disposition of formulated oligonucleotides have been proposed by the Drug Information Association affiliated Oligonucleotide Safety Working Group (22–27) and these papers provide helpful information on industry best practices, although they are not considered a ‘guidance’ by regulatory authorities.

Table 2 summarizes the approval packages of all siRNA therapeutics approved to date. The timeline illustrates how approval packages are evolving as experience is gained with this modality. For example, neither the patisiran nor givosiran filing packages mention bioanalytical measurement or ADME properties of the sense strand. Furthermore, for givosiran radiolabeled ADME studies were conducted with [^3^H]-siRNA, whereas the corresponding studies for lumasiran and inclisiran utilized [^14^C]-siRNA; while for patisiran [^14^C]-LNPs were used. In general, there appears to be emphasis on understanding the safety implications of parent drug accumulation, off-target liabilities, and formulation toxicities. PPB and DDI reports were included in all four approval packages albeit variable in design as will be discussed in this review article.

**Table 2.** Approval packages for siRNA approved for human use as of May 2021

Section	Sub-section	*In vitro*/ *in vivo*	Patisiran (2018)^a^	Givosiran (2019)^b^	Lumasiran (2020)^c^	Inclisiran (2020)^d^
SiRNA format and chemical modifications			LNP 2′*O*-methyl RNA modifications, DNA (dT) and DLin-MC3-DMA, PEG_2000_-C-DMG, DSPC and cholesterol	GalNAc–siRNA 2′*O*-methyl 2′-fluoro and phosphorothioate RNA modifications	GalNAc–siRNA 2′*O*-methyl 2′-fluoro and phosphorothioate RNA modifications	GalNAc–siRNA 2′*O*-methyl 2′-fluoro and phosphorothioate RNA modifications, DNA (dT)
Disease indication			polyneuropathy of hereditary transthyretin-mediated amyloidosis	acute hepatic porphyria	primary hyperoxaluria type I	primary hypercholesterolaemia (heterozygous familial and non-familial) or mixed dyslipidaemia
Non-clinical pharmacology	Primary pharmacology	*in vitro*	target and off-target binding hepatocyte efficacy (human and monkey)	hep3B efficacy (human) SNP population profile of mRNA target (*in silico*) AS 3′(*N*– 1) metabolite hep3B	hepatocyte efficacy (monkey)	SNP population profile of mRNA target (*in silico*)
		*in vivo*	efficacy and duration (transgenic mouse, monkey)	efficacy and duration in WT (rat, monkey) efficacy and duration in disease models (mouse, rat)	efficacy and duration in WT (mouse, rat, monkey) efficacy and duration in disease models (mouse, rat)	efficacy and duration (transgenic mice, rat, monkey)
	Secondary Pharmacology	*in vitro*	none	activity with off-target RNA	activity with off-target RNA	activity with off-target RNA
		*in vivo*	off-target activity (serum retinol binding protein)	not included	not included	not included
	Safety pharmacology	*in vitro*	hERG conductivity (LNP)	not included	not included	not included
		*in vivo*	cardiovascular, respiratory, CNS (monkey)	cardiovascular, respiratory, CNS (monkey)	cardiovascular, respiratory, CNS (monkey)	cardiovascular, respiratory, neurological (monkey)
	Pharmacodynamic DDI	*in vivo*	not included	not included	not included	co-administration with atorvastatin (monkey)
Non-clinical ADME/PK	Absorption/PK	*in vivo*	plasma, spleen and liver PK (rat, monkey)	plasma, liver and kidney PK of parent. Plasma PK of AS 3′(N-1) metabolite (rat, monkey)	plasma and liver PK of parent (rat and monkey) s.c. bioavailability (rat, monkey)	plasma and liver PK of parent (rat and monkey) s.c. bioavailability (rat, monkey)
	Distribution	*in vitro*	albumin, α1-acid glycoprotein binding (rat, human)	PPB (mouse, rat, monkey, human) bioavailability (rat, monkey)	PPB (rat, monkey and human)	PPB (mouse, rat, monkey and human)
		*in vivo*	^14^C QWBA (rat), ^14^C ADME (labeled on LNP component; rat, monkey) PK (rat, monkey)	tissue distribution parent (rat) parent and AS 3′(N-1) metabolite (mouse) tissue distribution (pregnant rats, pregnant rabbits) ^3^H QWBA (rat) PK (mouse, rat, monkey) bioavailability (rat)	^14^C QWBA (rat) PK (rat, monkey)	^14^C QWBA (rat, monkey) tissue distribution (rat, monkey) PK (rat, monkey)
	Metabolism	*in vitro*	met ID) serum and liver S9, fraction (mouse, rat, monkey, human) met ID recombinant CYP (human) inhibition/induction: CYPs, UGT1A1, transporters	met ID serum and liver S9 fraction (mouse, rat, monkey, human) lack of NADPH dependency in human liver S9 fractions DDI (transporter substrate/inhibition, CYP substrate/inhibition/induction)	serum stability (mouse, rat, monkey, human) +/human liver S9 fraction met ID plasma and liver S9 fraction (mouse, rat, monkey, human) met ID human hepatocytes DDI (CYP substrate/inhibition/TDI)	serum stability (mouse, rat, monkey, human) met ID serum and liver S9 fraction (mouse, rat, monkey, human) DDI (transporter substrate/inhibition, CYP inhibition/induction/TDI)
		*in vivo*	met ID (rat, monkey, human)	unlabeled and ^3^H met ID in intact and BDC animals (rat) met ID in plasma and urine (monkey, human)	unlabeled and ^14^C met ID in intact and BDC animals (rat, monkey) met ID plasma and urine (human)	unlabeled and ^14^C (rat, monkey) met ID (human)
	Excretion	*in vivo*	^14^C excretion mass balance (rat, monkey)	^3^H excretion mass balance in intact and BDC animals (rat) seminal fluid (rabbit) milk (rat)	-^14^C excretion mass balance (rat, monkey) seminal fluid (rabbit)	^14^C excretion mass balance (rat, monkey) milk (rat)
Non-clinical toxicology	Toxicokinetics species		rat, monkey	mouse, rat, monkey	rat, monkey	mouse, rat, rabbit, monkey
	Genotoxicity	*in vitro*	bacterial reverse mutation Ames test chromosome aberration	bacterial reverse mutation Ames test chromosome aberration	bacterial reverse mutation Ames test chromosome aberration	Ames test chromosome aberration
		*in vivo*	micronucleus test (mouse)	micronucleus test (rat)	micronucleus test (rat)	micronucleus test (rat)
	Carcinogenicity		long term studies (transgenic mouse)	long term studies (transgenic mouse)	long and short-term studies (transgenic mouse, rat)	long and short-term studies (transgenic mouse, rat)
	Reproductive and developmental toxicology	*in vivo*	fertility (rat) and embryo development (rat, rabbit) prenatal and postnatal development (rat)	fertility and embryo development (rat, rabbit) prenatal and postnatal development (rat)	fertility and embryo development (rat, rabbit) prenatal and postnatal development (rat) placental transfer juvenile toxicity (rat)	fertility and embryo development (rat) prenatal and postnatal development (rat)
	Other		human blood hemolysis (*in vitro*) immunogenicity and stimulation (rat, monkey)	ADA (rat, monkey)	renal impairment (rat) ADA (rat, monkey) impurity toxicology (rat)	none
Clinical	Other	*in vivo*		CYP perpetrator DDI hepatic and renal impairment		hepatic and renal impairment

Abbreviations: ADA, anti-drug antibodies; AS, antisense strand; BDC, bile duct cannulation; CNS, central nervous system; CYP, cytochrome P450; DDI, drug–drug interaction; DSPC, 1,2-distearoyl-*sn*-glycero-3-phosphocholine; EMA, European Medicine Agency; FDA, Food and Drug Administration; hERG, human Ether-a-go-go; HSPC, l-α-phosphatidylcholine; LNP, lipid nanoparticle; Met ID, metabolite identification; PEG-DMG, 1,2-dimyristoyl-*rac*-glycero-3methoxypolyethelene glycol; PK, pharmacokinetics; PPB, plasma protein binding; QWBA, quantitative whole body autoradiation; s.c., subcutaneous; SNP, single nucleotide polymorphism; TDI, time-dependent inhibition.

^a^Patisiran FDA and EMA filings.

^b^Givosiran FDA and EMA filings.

^c^Lumasiran FDA and EMA filings.

^d^Inclisiran EMA filing.

### PPB and DDI assessment in regulatory filing

Although PPB and DDI assessments have typically been included in siRNA regulatory filing packages in a similar manner and in line with small molecule filing, the field lacks clarity on whether such evaluations are relevant or necessary for this modality. In this publication, we comprehensively review publicly available siRNA PPB and DDI data (Parts 1 and 2, respectively). We systematically address the findings and critically question how the data may help to inform safety and efficacy to aid in human dose prediction, clinical development planning, and labeling. This information is summarized into two decision trees (Figures 3 and 4), which we propose as future recommendations to guide the discovery and development of siRNA therapeutics (Part 3).

**Figure 2.**
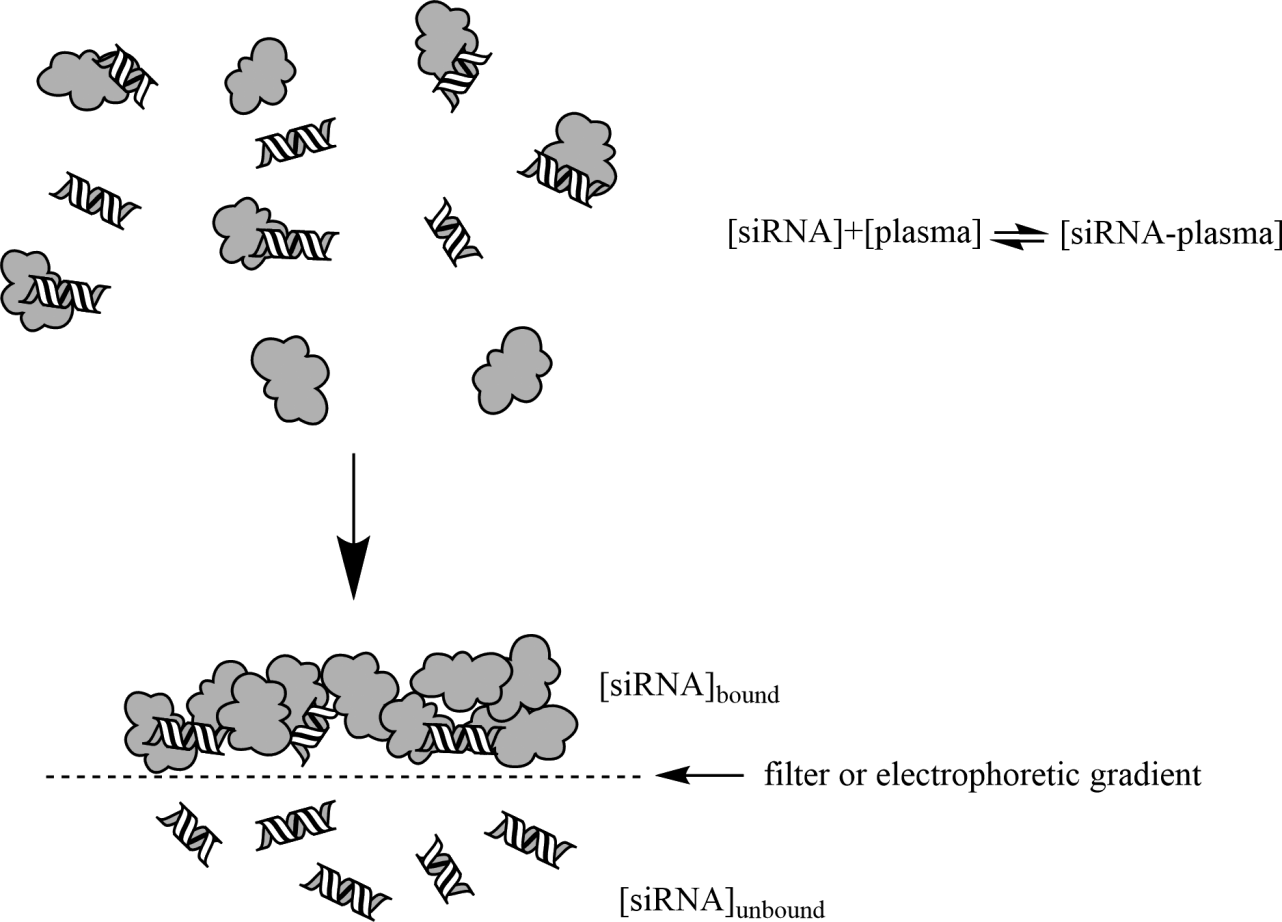
siRNA plasma protein binding is defined as the separation of the plasma protein bound fraction from the unbound fraction at equilibrium. Percent PPB is calculated as the bound concentration divided by the total concentration multiplied by one hundred.

**Figure 3.**
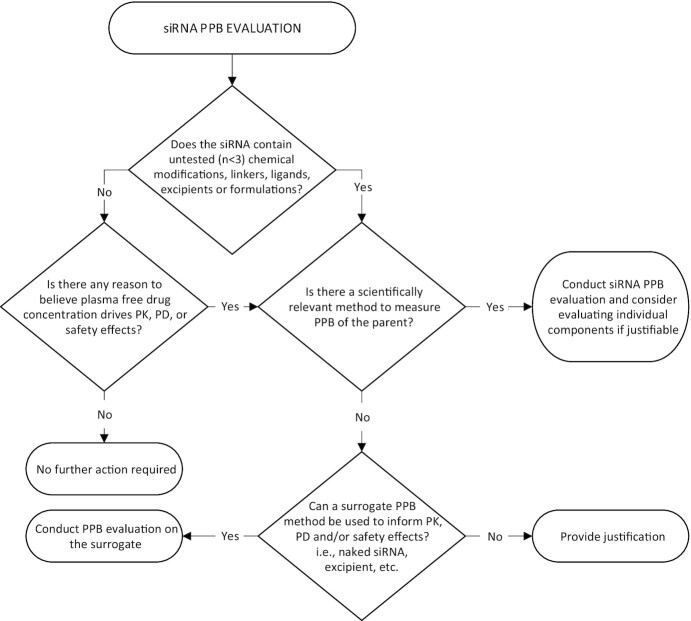
Recommended decision tree for siRNA PPB evaluation. Abbreviations: siRNA, short-interfering RNA; PPB, plasma protein binding.

### PART 1: siRNA PLASMA PROTEIN BINDING

Plasma protein binding is defined as the fraction of drug bound to proteins in plasma at equilibrium (Figure 2). From a modality perspective, while small molecule drugs exhibit a wide range of PPB that is closely linked to their ADME and PK/PD properties and therapeutic index (28–31), large molecule biologics, such as mAbs, are not routinely screened for PPB, as it is not anticipated to drive disposition and/or efficacy. Although it is less well-characterized, siRNA PPB is somewhat intermediary to these two extremes. Published studies addressing the extent and variability of siRNA PPB binding, and its role in PK are limited (32–34). In this section, we address the bioanalytical challenges of measuring siRNA PPB, review PPB data in regulatory approval documents, discuss the effects of PPB in ADME and PK/PD, and summarize safety concerns relating to PPB.

### siRNA PPB methodology

*In vitro* small molecule PPB is routinely measured during drug discovery and translation using various techniques including equilibrium dialysis, ultracentrifugation and ultrafiltration (28). The physicochemical properties of siRNA deviate significantly from small molecule, notably in terms of molecular weight, shape, and surface charge. Consequently, direct application of small molecule PPB methods for determination of siRNA PPB result in recovery issues (33,34). Instead, quantification of the unbound fraction of siRNA in plasma has required adaption of existing methods, such as the ultrafiltration method developed by Humphreys *et al.* (33), or development of alternative approaches, such as the electrophoretic mobility shift assay (EMSA) developed by Rocca *et al.* (34). The ultrafiltration method uses a plate-based hybridization immunoassay for bioanalytical quantitation with a lower limit of detection in the picomolar range (35). The EMSA method quantifies binding by comparing the fluorescence intensity of the free siRNA band in the presence and absence of plasma in a 2D polyacrylamide gel stained with an intercalating dye; the lower limit of detection is not reported. To date, only EMSA-derived PPB has been reported for approved GalNac–siRNA molecules.

Challenges exist with both the ultrafiltration and EMSA approaches to measure the siRNA PPB at equilibrium, and a head-to-head evaluation is needed to establish best practices. The ultrafiltration method requires a 50 kDa molecular weight cut-off filter to enable adequate recovery and separation of the free fraction from the bound since the hydrodynamic radius of the siRNA polymer is slightly less than a globular 50 kDa protein. Consequently, if siRNA is bound to plasma proteins smaller than ∼35 kDa, this complex may be reported as part of the free fraction. This method also encounters recovery issues that can be addressed via pre-treatment of surfaces with detergent such as Tween-20 or CHAPS at low concentration (0.1% (w/v)), which may modify protein binding. Furthermore, while this method has been applied to GalNAc-conjugated siRNA, it may require further empirical development for use with other conjugates with respect to recovery. Due to the large hydrodynamic radius of LNPs (>>50 kDa globular protein equivalent), PPB by ultrafiltration is not compatible with that siRNA delivery format (33). The EMSA approach requires dilution steps into PBS and EMSA gel loading solution, both of which represent a perturbation of the equilibrium prior to measurement. In addition, given that separation by EMSA occurs over minutes, this too, could alter the partitioning of siRNA between bound and free states. PPB for LNPs was not reported using the EMSA method and full bioanalytical method validation for the ultrafiltration and EMSA methods are not publicly available.

Other approaches employed to qualitatively assess binding of siRNA to plasma include surface plasmon resonance (34), biolayer interferometry (33), and fast protein liquid chromatography followed by SDS-PAGE (EMA Assessment Report (EMA/554262/2018)). Of these methods, biolayer interferometry has also been used to assess binding to specific individual plasma proteins, including albumin, alpha-2-macroglobulin, fibrinogen and thrombin (33).

### siRNA PPB in regulatory filings

In the absence of a PPB guidance for siRNA, nonclinical safety studies typically follow the guidance for small molecule (ICH M3(R2)) however, specific studies can be omitted or added on a case-by-case basis. All approved siRNA as of May 2021 reported PPB in their filings, as summarized in Table 3. It should be noted that patisiran is the only approved siRNA formulated as an LNP, and that, regarding PPB, the European Public Assessment Report states that ‘the accuracy of the results was difficult to determine due to the assay used’ (EMA/554262/2018). Patisiran was a first in class therapeutic and the standard assays for small molecule PPB were not applicable, highlighting the fact that measuring PPB of the parent drug can be technically challenging.

**Table 3.** siRNA plasma protein binding reported in regulatory filings up until 2020

	Patisiran^a^	Givosiran^a^	Lumasiran^a^	Inclisiran^a^
**Species tested**	unknown	mouse, rat, monkey, human	mouse, rat, monkey, human	mouse, rat, monkey, human
**C_max_ (human therapeutic dose)**	7050 ng/ml (0.3 mg for patients < 100 kg, 30 mg for patients > 100 kg by i.v. infusion)	321 ng/ml (2.5 mg/kg by s.c. injection)	701 ng/ml (3–6 mg/kg (based on body weight) with loading doses by s.c. injection)	507 ng/ml (284 mg initially, at 3 months, then every 6 months thereafter)
**Delivery vehicle**	LNP	GalNAc conjugate	GalNAc conjugate	GalNAc conjugate
**Concentration tested**	unknown	0.5–50 μg/ml (EMA) 1–50 μg /ml (FDA)	0.5–50 μg/ml (EMA) 5–100 μg/ml (FDA)	0.5 μg/ml (human C_max_)
**PPB (across concentrations tested)**	∼97%^b^	10–91% (mouse) 28–93% (rat) 26–90% (monkey) 21–92% (human)	35–96% (rat) 37–86% (monkey) 20–85% (human) (EMA) FDA^d^	87% (human) < (mouse, monkey) < 93% (rat)
**Human PPB at clinically relevant concentrations**		92% at 1 μg/ml	77–85% at 0.5–1 μg/ml	87% at 0.5 μg/ml
**Specific siRNA-plasma protein interactions**	Rat serum albumin – 0.89%^c^ Human serum albumin – 0.46%^c^ Human a1-acid-glycoprotein – 2.1%^c^			
**Method**	PPB – not reported Specific plasma protein interactions – FPLC followed by SDS-PAGE	EMSA	EMSA	Not reported

Abbreviations: C_max_, maximum concentration in plasma; EMA, European Medicine Agency; EMSA, electrophoretic mobility shift assay; FDA, Food and Drug Administration; GalNAc, *N*-acetylgalactosamine; LNP, lipid nanoparticle; PPB, plasma protein binding.

^a^Patisiran, givosiran and lumasiran data are from combined FDA and EMA approval documents. Inclisiran data is from EMA approval document. All PPB values reported here have been rounded to two significant figures.

^b^Reflects binding of the lipid excipient only (no siRNA) to human plasma.

^c^Reflects binding of intact LNP to rat serum albumin, human serum albumin and human alpha-1-acid glycoprotein separately.

^d^EMA results should be used for this assay after discussion with Alnylam.

To date, GalNAc–siRNA PPB values, where reported, exhibit concentration dependence across species with higher PPB at lower concentrations (givosiran and lumasiran). There does not appear to be a significant difference between species (33). At therapeutically relevant concentrations (C_max_ at the human therapeutic dose), PPB ranges from approximately 77% to 92% (FDA Multi-discipline Review (NDA 212194), FDA Integrated Review (NDA 21410)).

### Effect of siRNA PPB on ADME and PK/PD

The significance of PPB for small molecules is closely related to the widely accepted free drug hypothesis, which is comprised of two parts: (i) free drug concentration at the site of action is what drives pharmacological effects and (ii) at steady state, the free drug concentration is the same at both sides of any biological membranes for compounds that are not substrates for uptake or efflux transporters (29,36). The second part is particularly important for PK/PD understanding of small molecule drugs modulating intracellular targets. Therefore, PPB is a critical parameter that is routinely measured for small molecule drugs for determination of PK/PD relationships, cross-species translation, and therapeutic index calculations. In contrast, it is widely accepted that PPB studies or data are not needed for antibody therapeutics as free drug is typically directly measured and the second part of the free drug hypothesis does not apply since mAb targets are extracellular. For siRNA, given that dosing frequency is monthly or longer, it is not membrane permeable, and it is in circulation only transiently (a few hours) yet it is sustained in tissue—plasma and target tissue steady-state levels are disconnected, so the second part of the free drug hypothesis does not apply. Consequently, PPB has little to no impact on characterizing PK/PD relationships. Here we review the current body of data from regulatory filings pertaining to the role of siRNA PPB on its PK and PK/PD properties, shedding additional light on the lack of utility of this measurement. Given their divergent physicochemical properties, LNP siRNA and GalNAc-conjugated siRNA are discussed separately.

#### Effect of PPB on pharmacokinetics of LNP formulated siRNA

Patisiran was the first and only approved siRNA therapeutic that is formulated as an LNP (as of May 2021). It was approved for treatment of patients with hereditary transthyretin-mediated amyloidosis and consists of a 21-mer double-stranded siRNA (ALN-18328) encapsulated in four lipid excipients. Patisiran is administered at 0.3 mg/kg every 3 weeks by intravenous (i.v.) infusion. The plasma PK profile of the ALN-18328 component is characterized by an initial steep distribution phase with an estimated *t*_1/2α_ of ∼1 h, then a minor second peak, followed by a relatively long terminal phase with an estimated *t*_1/2β_ of ∼3 days. ALN-18328 remains encapsulated within the LNP in circulation and is cleared through LNP-mediated liver uptake. Once in the liver, a fraction of ALN-18328 dissociates from the nanoparticle and binds to RISC to exert its pharmacological effect; the rest remains encapsulated and is redistributed back into circulation, likely through exocytosis. Once ALN-18328 is released from the nanoparticle it is rapidly cleared, likely through metabolism by serum and tissue exonucleases. Less than 1% of the parent ALN-18328 is excreted in the urine (37).

For patisiran (ALN-18328 + LNP’s PEG excipients), binding to plasma proteins was reported in two different ways. Binding of patisiran was assessed in rat serum albumin, human serum albumin and human alpha-1-acid glycoprotein, and was found to be 0.89%, 0.46% and 2.07%, respectively. In addition, binding to total human plasma was measured for the PEG2000-C-DMG alone and determined to be ∼97% (FDA Multi-discipline Review (NDA 210922)). Although these data were included in the New Drug Application filing, they were not used for any ADME or PK/PD interpretation or cross-species translation. Free non-encapsulated ALN-18328 accounted for <5% of circulating ALN-18328 and no PPB values were reported for ALN-18328 itself. The lack of observable impact of PPB on the disposition of patisiran may or may not be applicable to other siRNA therapeutics that are formulated as stable nanoparticles to facilitate tissue uptake. However, the impact will depend on the nature of the nanoparticle (if highly protein bound then more likely to have an impact) and whether PPB impacts downstream tissue uptake.

#### Effect of PPB on pharmacokinetics of GalNAc-conjugated siRNA

Three out of the four approved siRNA therapeutics are GalNAc-conjugated siRNA (givosiran, lumasiran, and inclisiran) delivered via subcutaneous (s.c.) injection. Similar to the strong class effect of GalNAc-conjugated ASOs on pharmacokinetics (38,39), the pharmacokinetics of GalNAc-conjugated siRNA therapeutics also appear to be similar between molecules and are highly predictable and scalable across species (10). In humans, GalNAc–siRNA exhibit a plasma *T*_max_ of ∼4–6 h, elimination half-lives of ∼5–10 h, and apparent systemic clearance of ∼27–38 L/h in healthy subjects. The majority of the administered dose is taken up by the parenchymal liver cells and metabolized by tissue-localized endo- and exonucleases. Renal excretion (parent and metabolites combined) accounts for 15–20% of systemic clearance at doses administered clinically.

Within the therapeutic dose range, PPB was measured to be >90% for givosiran, 77–85% for lumasiran and ∼87% for inclisiran (FDA Multi-discipline Review (NDA 212194), EMA Assessment Report (EMA/CHMP/70703/202), Integrated Review (NDA 21410), EMA Assessment Report (EMA/568312/2020), EMA Assessment Report (EMA/696912/2020)). While these values were included in regulatory application packages, they were not utilized to increase understanding of ADME characteristics and PK/PD relationships due to lack of any observable impact of PPB on the ASGPR-mediated hepatocellular uptake, disposition or PK/PD properties of these GalNAc–siRNA (32).

From a pharmacokinetics perspective, the systemic clearance of these molecules is mediated by ASGPR-mediated liver uptake, accounting for a majority of total clearance (EMA Assessment Report (EMA/696912/2020), (10,40)). Therefore, it is reasonable to postulate that the GalNAc ligand is the primary driver of liver uptake efficiency. This hypothesis is supported by data demonstrating that the dose-normalized plasma area-under-the-curve (AUC) are similar between GalNAc-conjugated siRNA and GalNAc-conjugated ASOs, which tend to be substantially more protein bound than siRNA (D. Ramsden, 22nd Annual Drug Metabolism and Applied Pharmacokinetics Conference, ‘*Leveraging the unique ADME properties of GalNAc–siRNA: Not a small or large molecule*.’ Madison, Wisconsin, USA (2019)). Furthermore, Agarwal *et al.* (32) recently demonstrated that serum proteins have minimal impact on GalNAc–siRNA and GalNAc–ASO uptake and activity in human hepatocytes.

Renal clearance is a relatively minor pathway for elimination of GalNAc-conjugated siRNA, accounting for < 15% of human systemic clearance for givosiran, ∼16% for inclisiran and ∼20% for lumasiran (EMA Assessment Report (EMA/CHMP/70703/2020), EMA Assessment Report (EMA/568312/2020)). For these three GalNAc–siRNA the rate of renal clearance is close to the total glomerular filtration rate without accounting for the unbound fraction in plasma. For inclisiran a 2.3-fold increase in plasma exposure has been observed in patients with severe renal impairment. The renal clearance without correction for PPB of inclisiran was on average 5.6, 3.7, 1.5 and 0.5 L/h in subjects with normal renal function, mild, moderate and severe renal impairment, respectively, which is in line with the glomerular filtration rate in these subjects (41). Overall, these results suggest that PPB does not effectively protect GalNAc-conjugated siRNAs from glomerular filtration. For unconjugated ASOs, chemical modifications (e.g. phosphorothioate backbone) designed to increase PPB (primarily through albumin binding) have been shown to effectively protect ASOs from renal filtration (42). Two recent studies investigating PPB of siRNA demonstrated that GalNAc-conjugated siRNAs do not bind to albumin (28,29) but can bind to α-2-macroglobulin (720 kDa), α-thrombin (37 kDa), fibrinogen (340 kDa), and fibronectin (500 kDa), all of which do not undergo glomerular filtration. This suggests that binding between GalNAc–siRNAs and these proteins may not be tight enough to prevent renal filtration.

#### Effect of PPB on PK/PD understanding of siRNA therapeutics

A prominent feature of the PK/PD relationship for siRNA therapeutics is the apparent disconnect between the transient plasma exposure and the prolonged pharmacological effects. For all approved siRNA therapeutics, whether LNP-formulated or GalNAc-conjugated, plasma exposure typically declines below detection limits within days while the pharmacological effect endures for weeks to months. In fact, inclisiran remains active >6 months post dose. This extended pharmacodynamic (PD) durability is driven in part by sequestration in hepatocyte endosomes, enhanced metabolic stability, slow endosomal release resulting in prolonged RISC loading, and the autocatalytic nature of RNA silencing in tissues resulting in improved liver exposure and prolonged tissue half-lives (43).

Similar to small molecule drugs, the pharmacological effect of siRNA is driven by the free drug at the site of action, which is the RISC complex in the cytosol of the targeted cells. The pharmacological effect of current generation liver-targeting siRNA therapeutics starts with hepatic uptake via LNP (i.e. patisiran) or GalNAc-mediated pathways, neither of which appear to be affected by PPB. Once in the liver, the free drug concentration in the cytosol depends on endo-lysosomal drug stability and escape efficiency, liver metabolic stability, and cytosolic protein binding, not PPB. Since siRNA are only present transiently in the plasma, they are not membrane permeable, and they are drawn into the cell via active uptake, an equilibrium will not be established across the hepatocyte membrane. Therefore, PPB data has no appreciable value for PK/PD understanding, for either LNP or GalNAc-delivered siRNA.

Although no lipid-conjugated siRNA have been approved for human use, they have been studied extensively in the preclinical space. Lipid–siRNA conjugates can have extensive protein binding that may lead to significant differences in the biodistribution and PK of these molecules (44–47). While it is conceivable that lipid-siRNA conjugates or other conjugates with a high affinity for plasma proteins may have an extended plasma half-life, to our knowledge, the extent of PPB at therapeutically relevant concentrations has not been reported for these molecules, and the role of the plasma as a depot to drive PD effects is not well understood.

### siRNA PPB safety considerations

Severe thrombocytopenia was observed in the clinic for two ASOs (Ionis-TTRRx and Ionis-ApoCIIIRx) and peripheral neuropathy was observed for one siRNA (revusiran; discontinued). While the mechanisms for these adverse events have not yet been fully explained, it is plausible that they could be C_max_ driven or associated with protein binding (48). An Oligonucleotide Safety Working Group white paper by Henry *et al.* (27) documents the role of oligonucleotides in activation of complement in plasma by the alternative pathway in a concentration-dependent manner in monkeys. Early generations of ASOs encountered acute, transient, alternative activation of complement that was largely attributed to non-specific protein binding driven by the high phosphorothioate content (49–51). However, the effect may be less prominent in humans (52). Compared to ASOs, siRNA typically have minimal phosphorothioate content and lower PPB (33). In cases where complement activation by siRNA have been observed, it was purportedly related to the delivery of cationic lipid-containing formulation excipients, rather than the oligonucleotide itself, and occurring via the classical pathway rather than the alternative pathway (27).

### PART 2: siRNA DRUG–DRUG INTERACTIONS

Drug–drug interactions are caused by a mutual physiological interaction between at least two co-administered drugs. In this context, co-administered drugs are considered as xenobiotics that have the potential to affect the ADME and/or efficacy of one another. While dietary compound-drug interactions and drug-endogenous compound interactions are discussed in the literature, the current section will focus on siRNA interactions with other therapeutics. Although FDA guidance does not specifically mention siRNA or ONTs, it emphasizes evaluating DDIs both preclinically and clinically.

SM *in vitro* DDI workflows focus on major known drug metabolizing enzymes and transporters primarily expressed in the liver and kidney. Thorough reaction phenotyping is recommended to identify metabolic contributors among the phase I and phase II enzymes, as well as transporters. In addition to phenotyping, studies to assess the DDI potential of the small molecule as a victim (substrate), perpetrator (inhibitor) and inducer are also required. Regulatory guidance from the EMA, FDA, and Japanese Pharmaceutical Medicines Device Agency require standardized *in vitro* assays to determine whether clinical DDI studies are needed (https://www.ema.europa.eu/en/documents/scientific-guideline/guideline-investigation-drug-interactions-revision-1_en.pdf; https://www.fda.gov/media/134581/download; https://www.fda.gov/media/134582/download; https://www.pmda.go.jp/files/000228122.pdf; (53)). For therapeutic proteins, DDI regulatory guidance is mainly concerned with the pharmacological impact of the drug. Cytokine modulating therapies, for example, may up- or down-regulate cytochrome P450s (CYPs) and cause DDIs indirectly. However, DDIs with therapeutic proteins are rare and typically weak if observed (54).

At the time of writing there is no literature precedent suggesting that siRNA will compete with co-administered drugs for drug metabolizing enzyme or transporter active sites at therapeutically relevant concentrations. Therefore, the potential DDI landscape for siRNA is likely to include mechanism-based effects or disease drug interactions. In this section, we discuss siRNA DDI study design, direct inhibition and induction interactions of CYPs and transporters, mechanism-based interactions, drug disease interactions, and review the regulatory filings concerning siRNA DDI evaluations.

### siRNA DDI study design considerations

Differences in size, physicochemical and ADME properties between siRNA and small molecules mean that siRNA DDI study protocols cannot be directly adapted from SM. Practical experimental design considerations such as siRNA concentration, protein matrices, incubation times, and special considerations for alternative siRNA formats are necessary for thorough preclinical testing.

Selecting an siRNA concentration range for *in vitro* DDI studies is complicated by factors such as rapid systemic clearance and non-oral delivery routes. Since siRNA clears rapidly from the blood, the small molecule rationale of using plasma C_max_ at therapeutically relevant concentrations as a benchmark for experimental design is not relevant as it would likely overestimate *in vivo* DDI potency. The hepatocyte-targeting behavior of GalNAc–siRNA and certain LNPs including patisiran results in significant distribution of the drug to the liver compared to the plasma. Furthermore, intracellular compartmentalization due to the mechanism of uptake may decrease the free drug available to interact. To account for a ‘worst case scenario,’ although cumbersome, the EMA recommends using the predicted human liver concentration from preclinical species for DDI assessment (Guideline on the investigation of drug interactions (CPMP/EWP/560/95/Rev. 1 Corr. 2; https://www.ema.europa.eu/en/documents/scientific-guideline/guideline-investigation-drug-interactions-revision-1_en.pdf)). *In silico* simulation tools, such as PK simulations and allometric scaling of liver concentrations from preclinical species to humans as described by Ramsden et al. (55), are necessary to estimate the appropriate concentration of siRNA for *in vitro* study design. Although typically considered negligible, direct measurement of fraction unbound in common *in vitro* matrices such as microsomes or hepatocytes may be useful for *in vitro–in vivo* correlations (33,55).

Since therapeutic GalNAc–siRNA are relatively stable in liver *in vivo*, these compounds are anticipated to undergo minimal nucleolytic cleavage *in vitro* (17). The incubation timing aspect of *in vitro* DDI assays should therefore be modified from typical small molecule methods. Experiments to evaluate metabolism over longer incubations (such as for testing time-dependent inhibition) need to be designed with an understanding of the stability of the specific siRNA(s) being tested and the half-life of the enzyme affected.

Although it is common to compare DDI readouts for small molecule drugs in different *in vitro* assay systems such as primary hepatocytes and microsomes to enable differentiation between activities of cytosolic and membrane-associated drug metabolizing enzymes, such an approach is not feasible or relevant for GalNAc–siRNA, for example, Brown et al. (9) use cellular distribution studies to demonstrate that GalNAc–siRNA is taken up into primary hepatocytes very slowly via free uptake by ASGPR-mediated endocytosis over 7–24 h, compared to transfection at <7 h. The GalNAc–siRNA that is absorbed via free uptake is primarily localized in acidic compartments such as endosomes and lysosomes. Since transfection is physiologically irrelevant from a drug delivery perspective, and most drug metabolizing enzymes such as CYPs are found in the cytosol and in cytosol-facing membranes, siRNA DDI measurements (i.e. enzyme activity and mRNA level changes) by free uptake in hepatocytes are likely hindered by the siRNA cellular distribution profile. As such, hepatocyte assays (at least using conventional small molecule assay conditions) are not an appropriate *in vitro* system for siRNA evaluation. Furthermore, this highlights that any DDIs identified with siRNAs using microsomes may be overestimating the effect since siRNA is found in low abundance in the endoplasmic reticulum-facing compartment.

Despite efforts to optimize methods to evaluate direct DDIs between siRNA and SM driven by siRNA interactions with drug metabolizing enzymes and transporters, there is little evidence to suggest such DDIs are anticipated. With the current generation of siRNA, the greatest DDI risk is likely to arise from the PD response. Additional care should be taken to examine down-stream effects of the intended behavior of any siRNA therapeutic, and design specific *in vitro* DDI studies with PD endpoints in mind where *in vitro* studies are relevant.

### Direct CYP inhibition and induction siRNA DDIs

The major breakdown or catabolic pathways of unconjugated siRNA are via nuclease-mediated hydrolysis. Thus, siRNA are not anticipated to be CYP substrates. To date, no CYP-mediated metabolites have been reported for any of the approved siRNA. Further, comprehensive *in vitro* phenotyping using cDNA-expressed isozymes indicated that GalNAc–siRNA are not substrates of CYP1A2, 2B6, 2C8, 2C9, 2C19, 2D6, 3A4 or 3A5 mediated clearance (EMA Assessment Report (EMA/696912/2020)). The lack of CYP involvement in the metabolism of siRNA is consistent with the physicochemical properties of siRNA, such as high molecular weight, hydrophilicity, and polyanionic nature. Due to their lack of affinity toward CYP enzymes, siRNA do not result in DDIs with other SMs which are CYP substrates.

Ramsden *et al.* (55) published an overview of *in vitro* DDI studies conducted for multiple GalNAc–siRNAs including givosiran, lumasiran, and inclisiran. CYP inhibition (reversible and time-dependent) evaluations were performed using pooled human liver microsomes as the test system. The 12 siRNA molecules evaluated showed no reversible or time-dependent inhibition of CYP1A2, 2C9, 2C19, 2D6 or 3A4/5. Three of the 12 siRNA showed direct inhibition of CYP2C8 at high concentrations with IC_50_ values of 224, 56 and 416 μM, which is >510-fold higher than the clinically relevant plasma C_max_. Cemdisiran also caused concentration dependent inhibition of CYP2B6 with an IC_50_ value of 583 μM, >12 000-fold higher than the observed clinical plasma C_max_. No time-dependent inhibition was observed for CYP2C8 or CYP2B6 by any of the GalNAc–siRNA molecules. An assessment of CYP induction potential of five GalNAc–siRNA molecules was performed using cryopreserved human hepatocytes from three different human donors as the test system. The results demonstrated no concentration-dependent or statistically significant increase in CYP metabolic activity or mRNA levels by any of the GalNAc–siRNAs evaluated. It was concluded that the totality of data supports that direct inhibition or induction of CYP enzymes by GalNAc–siRNA is highly unlikely at clinically relevant concentrations.

Of note, Ramsden *et al.* (55) did project that the clinical liver C_max_ for nine of the twelve GalNAc–siRNA ranged from 1.9–29 μM, which is 1.9–220-fold lower than the IC_50_ values observed for CYP2C8 inhibition. For the three approved GalNAc–siRNA, the projected clinical liver C_max_ is 3.2–58-fold lower. However, as stated by that group, there is no current guidance for establishing cut-offs in non-plasma matrices and the projections were based on exceedingly limited data.

### Direct transporter inhibition and induction siRNA DDIs

Drug transporters are a class of membrane-bound proteins that are expressed in various tissues and organs throughout the body. They often exhibit broad substrate specificity, and are involved in small molecule drug disposition. Both transporter substrates and inhibitors may affect PK/PD relationships when drugs are co-administered. Whether a drug binds to a drug transporter directly, or otherwise alters transporter activity or expression, a clinically relevant DDI may result. Transporter abundance and characterization at the molecular level have been established for a limited number of transporters for small molecule drugs; transporters specific to siRNA have not been described in the literature. Physiologically-based pharmacokinetic modeling of transporter-mediated DDIs may have utility to predict *in vivo* transporter-drug interactions, however, more work is needed (56). Regardless, these *in vitro* transporter assays were established for small molecule drugs, and their application to other modalities such as siRNA , is not appropriate and needs to be evaluated.

Ramsden et al. (55) also investigated the role of transporter interactions for 4 of the 12 GalNAc–siRNA molecules (including givosiran and inclisiran) using *in vitro* uptake (OAT1, OAT3, OATP1B1, OATP1B3, OCT1, OCT2) and efflux (BCRP, BSEP, MATE1, MATE2-K, P-gp) assays. Of the four siRNAs tested, none were substrates for transporters, while one (givosiran) caused inhibition of P-glycoprotein at concentrations that were well above clinically relevant levels. This implies that GalNAc–siRNA molecules are unlikely to be substrates for known small molecule drug transporters with low potential for inhibition. One likely explanation is that due to their large size, siRNA does not fit into the transporter binding sites. Although it is theoretically feasible that siRNA could block the entrance of the transporter binding sites or act as an allosteric inhibitor, *in vitro* transporter assays for siRNA molecules have not shown any interaction with transporter substrates.

### Direct mechanism-based interactions and indirect disease-drug interactions

While CYP and transporter inhibition and induction interactions are not anticipated for siRNAs, direct mechanism-based effects, indirect disease drug interactions, and direct inhibition and induction of siRNA-related proteins such as ASPGR and Ago2 should be considered. Direct mechanism-based effects are possible when an siRNA causes a PD effect that results in modulation of biochemical pathways regulating the expression of drug metabolizing enzymes such as CYPs and transporters.

Givosiran is an example of an approved siRNA that highlights the potential for PD driven DDIs. Givosiran modulates ALAS-1 mRNA transcript levels, and the ALAS-1 protein is the rate-limiting enzyme for heme biosynthesis in the liver. Consequently, givosiran decreases levels of the heme intermediates aminolevulinic acid and porphobilinogen in the liver. Decrease of these intermediates leads to reduction of hepatic heme content, and consequently to reduction in CYP enzyme levels and activities. Clinically meaningful DDIs were observed with givosiran and substrates of CYP1A2 and CYP2D6 resulting in a warning on the drug label stating that givosiran should not be used concomitantly with CYP1A2 or CYP2D6 substrates for which minimal concentration changes may lead to serious or life-threatening toxicities (FDA Multi-discipline Review (NDA 212194), EMA Assessment Report (EMA/CHMP/70703/2020), Prescribing Information (https://www.accessdata.fda.gov/drugsatfda_docs/label/2019/0212194s000lbl.pdf)).

Notably, any siRNA effects resulting in modulation of drug metabolizing enzyme expression and/or activity could potentially be prolonged due to the long duration of PD effect of siRNAs. This is especially important for co-administered drugs that have a low therapeutic index and are substrates for CYPs or drug transporters. The potential for DDIs mediated by mechanism-based effects should be evaluated for each siRNA on a case-by-case basis by understanding the upstream and downstream effects of target gene knockdown and/or predicted off-target silencing effects.

Certain inflammatory disease states including, but not limited to, influenza B, HIV infection, bone marrow transplant, sepsis, rheumatoid arthritis, and Crohn's disease, can result in clinically significant modulation of exposure of CYP and/or transporter-sensitive substrates (57–66). In such cases, the disease state reduces CYP and/or transporter activity or expression. Disease alleviation via treatment with siRNA may normalize or otherwise modulate the activity or expression, underscoring the importance of understanding the effect of reduction of disease on CYP and transporter function (67). As an example, this finding may be due to the direct mechanism-based effects of givosiran on heme biosynthesis as mentioned above, and/or by an indirect-disease drug interaction. Acute intermittent porphyria patients have been shown to have increased CYP expression and distinct CYP1A2 and CYP2D6 genotypes (68,69). The potential for DDIs mediated by indirect drug-disease based effects should be evaluated for each siRNA and target disease population.

Although there are no clinical reports of siRNA as a victim drug, in theory, if two GalNAc siRNA (or other GalNAc-conjugated species) were administered concomitantly, they could compete for ASGPR- mediated liver uptake or Ago2 in RISC. Furthermore, although published data is lacking, a perpetrator drug could conceivably modulate the expression or activity of ASGPR or Ago2 as a further example of potential mechanism-based interactions.

### Nonclinical and clinical investigations of CYP- and transporter-related DDIs for approved siRNA with co-administered drugs

Nonclinical *in vitro* DDI studies using liver microsomes were performed for all four approved siRNA therapeutics and clinical DDI studies were performed on givosiran and inclisiran due to the specific pharmacology of those drugs. These studies are summarized below.

#### Patisiran

*In vitro*, none of the LNP constituents induced CYP1A2 or 3A4 in human hepatocytes. CYP 2B6 showed a concentration dependent and >2-fold increase in mRNA levels upon treatment with patisiran; the effect *in vivo* has not been studied. CYPs 1A2, 2A6, 2B6, 2C8, 2C9, 2C19, 2D6 and 3A4/5 were not inhibited at therapeutically relevant levels. Patisiran was reported to be a CYP2B6 time-dependent inhibitor, but the *in vivo* effects are not established. The LNP constituents did not inhibit or alter the expression of human CYPs 1A1/2, 2C9, 2C19 2D6, or 3A or UGT1A1. Furthermore, the LNP constituents did not inhibit BCRP, MDR1, BSEP, OATP1B1, OATP1B3, OAT1, OAT3, OCT1, OCT2, MATE1 or MATE2-K. Transporter DDI liability was concluded to be minimal (EMA Assessment Report (EMA Assessment Report (EMA/554262/2018), FDA Multi-discipline Review (NDA 210922)).

#### Givosiran

*In vitro, ex vivo* and *in vivo* studies were conducted in the nonclinical and clinical settings. *In vitro*, givosiran was not a substrate, inhibitor, or inducer of CYPs or transporters; the CYPs and transporters evaluated were not disclosed. *Ex vivo and in vivo* DDI studies in rats and monkeys could not exclude indirect inhibition of CYPs via PD-based reduction of hepatic heme content, so a clinical study was warranted. The potential for a direct mechanism-based DDI by givosiran was investigated in a dedicated clinical DDI study to assess the interactions of givosiran with five major CYP enzymes (CYP1A2, 2C9, 2C19, 2D6 and 3A4). Clinically meaningful DDIs were observed with substrates of CYP1A2 (3× fold increase of caffeine AUC) and 2D6 (2.4× fold increase of dextromethorphan AUC). It remains an open question as to why *in vitro* to *in vivo* correlation for CYP1A2 and CYP2D6 was not observed for givosiran. In general complex processes such as heme-synthesis may not be fully reflected by cellular systems on the time-scale of *in vitro* experiments (FDA Multi-discipline Review (NDA 212194), EMA Assessment Report (EMA/CHMP/70703/2020)).

#### Lumasiran

Lumasiran was not found to be a substrate of CYP1A2, 2B6, 2C8, 2C9, 2C19, 2D6, 3A4 or 3A5. Reversible and time-dependent inhibition along with induction studies also yielded negative results, but the CYPs tested were not disclosed. *In vitro* transporter substrate/inhibition studies were not conducted, as it was deemed low risk based on aggregate data across similar GalNAc–siRNA conjugates (FDA Integrated Review (NDA 21410), EMA Assessment Report (EMA/568312/2020)).

#### Inclisiran

*In vitro*, inclisiran was not found to inhibit human CYP1A2, 2B6, 2C8. 2C9, 2C19, 2D6, 3A4/5, OAT1, OAT3, OCT1, OCT2, OCT3 OATP1B1, OATP1B3, MATE1, MATE2-K, MDR1, BSEP or BCRP at therapeutically relevant concentrations. For the CYPs, no time-dependent inhibition was observed, and inclisiran was not investigated as a CYP substrate. Inclisiran is not a substrate for OAT3, OCT1, OCT2, OCT3, OATP1B1, OATP1B3, MATE1, MATE2-K, MDR1, BSEP or BCRP; active transport of OAT1 could not be excluded. A DDI study in monkeys was conducted to evaluate the potential toxicity when inclisiran is combined with atorvastatin, another lipid-lowering drug that may be used in combination with inclisiran, and no toxicity was observed.

In two clinical studies, effect of statin on inclisiran PK and effect of inclisiran on statin PK was investigated. Based on data available, clinically meaningful interactions with atorvastatin, rosuvastatin or other statins are not anticipated (EMA Assessment Report (EMA/696912/2020)).

## PART 3: RECOMMENDATIONS FOR ASSESSMENT OF SIRNA PPB AND SIRNA DDIs TO SUPPORT REGULATORY FILING

### siRNA PPB assessment recommendations

The perspective of the IQ Consortium siRNA Working Group is that an siRNA PPB report should only be included in regulatory filings if the siRNA contains a novel chemical modification, linker, ligand, excipient, or formulation that hasn’t been tested in clinically approved drugs (*n* < 3 examples of similar approved drugs), and/or if there is reason to believe plasma free drug concentrations drive PK, PD and/or safety. The above examples from regulatory approval documents for all four siRNA approved to date demonstrate that PPB measurements have had little to no impact on safety margin determination. However, as the siRNA field is rapidly evolving, we recognize that this may not be the case for all siRNA moving forward. To this end, we have constructed a decision tree to aid industry researchers in determining whether siRNA PPB is necessary for their therapeutic candidates from a regulatory perspective (Figure 3).

Considering the low value of PPB to ADME and PK/PD understanding and to therapeutic index estimation, we recommend against inclusion of a PPB report in future regulatory filings for the current generation of GalNAc–siRNA. If there is clear evidence suggesting that a component or components of an siRNA-containing therapeutic candidate is likely to bind to plasma proteins, and that such binding could alter the PK, PD and/or safety margins, we recommend evaluating PPB of the parent drug or complex. Examples of ‘clear evidence’ might include when renal clearance is less than the glomerular filtration rate, plasma exposure is significantly higher than what might be expected for a typical GalNAc-siRNA, or when an siRNA is conjugated to a ligand or linker with a known or untested binding affinity for a plasma protein, i.e., lipid-conjugated siRNA binding to albumin. If PPB evaluation is not technically possible, we recommend utilizing a surrogate approach of evaluating PPB of individual components of the parent such as LNP excipients, naked siRNA, and ligands, with emphasis on components that are anticipated to directly interact with the plasma proteins (this may exclude naked siRNA that is encapsulated while in the plasma, for example). If a surrogate approach is not technically possible, or if it does not make scientific sense, justification is required.

Beyond the decision tree, we further recommend in cases where siRNA PPB evaluation is deemed necessary, that companies work towards publishing fit for purpose methods, and align on industry standard approaches where possible. Furthermore, while beyond the scope of this white paper, it may be of interest for companies to investigate the impact of protein binding on *in vitro* ADME assays in biological matrices that are more relevant to PK/PD for this modality. One example of many could be investigating the effect of protein binding using liver-derived matrices for GalNAc–siRNA.

### siRNA DDI assessment recommendations

**Figure 4.**
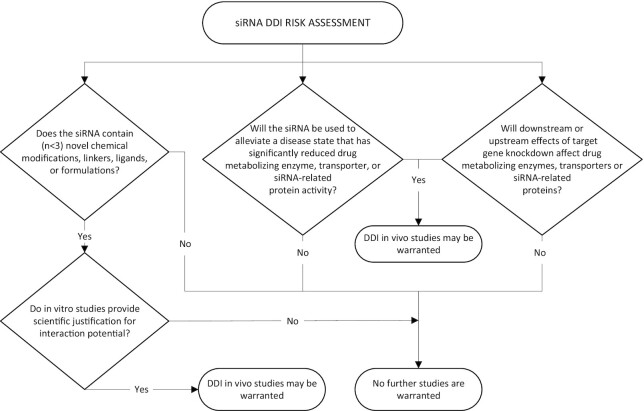
Recommended decision tree for siRNA DDI risk assessment. Abbreviations: DDI, drug–drug interaction; siRNA, short-interfering RNA.

The siRNA DDI risk assessment recommendations proposed in the DDI decision tree (Figure 4), and discussed below, are based on DDI guidance and literature for small molecule drugs and protein therapeutics, as well as published information on siRNA drugs approved to date by FDA and/or EMA. In deciding which drug metabolizing enzymes, transporters or other proteins should be included in the DDI evaluations for siRNAs, factors that should be taken into consideration include the chemical structure of the siRNA and any conjugates, the chemical nature of the excipients in the formulation, the target, the disease state and disease alleviation strategy, and any potential upstream or downstream effects of target gene knockdown on drug metabolizing enzymes, transporters or siRNA-related proteins (i.e. ASPGR for GalNAc–siRNA and Ago2).

In developing the proposed DDI decision tree, the primary considerations for assessing siRNA DDI potential were divided into three categories, with each category leading to a ‘yes or no’ decision for further evaluations. In the context of this discussion, the term ‘siRNA’ encompasses siRNAs delivered as LNP formulations, GalNAc–siRNA conjugates, siRNA-peptide or siRNA-protein conjugates, small molecule-siRNA conjugates, and any other siRNA-containing drug entities. We currently recommend *in vitro* assays be performed in liver microsomes, however, recognize that a cellular assay may have utility if free uptake can be demonstrated. Although we have made every effort to make these recommendations generalizable, we recognize the data that underpin the decision tree was largely derived from two siRNA platforms (LNP–siRNA and GalNAc–siRNA conjugates), and that it may need to be refined in future as new data come to light with other platforms.

The three categories for DDI assessments, and associated decision processes, are discussed below:

1. *Direct inhibition or induction of drug metabolizing enzymes, transporters or siRNA-related proteins*. One of the most important determinants for which drug metabolizing enzymes and drug transporters may be involved in the metabolism and transport of any particular drug is the chemical structure of the drug and/or the formulation in which it is delivered. Thus, the first question to consider for the siRNA DDI risk assessment is: Does the siRNA contain any novel chemical modifications, tertiary structures, or ligands, or does the siRNA formulation contain one or more novel excipients? Meaning classes of moieties that have been subjected to DDI risk assessment in a regulatory setting fewer than three times. If the answer is ‘no’, then no further DDI assessment is warranted. If the answer is ‘yes’, then relevant *in vitro* DDI studies, such as those described by Ramsden et al. (55), should be considered. If the *in vitro* data show low potential for *in vivo* DDIs, then *in vivo* DDI studies are not warranted. However, if the *in vitro* data indicate potential for *in vivo* DDIs based on the calculations included in the current regulatory guidance for small molecules (FDA DDI guidance 2017: https://www.fda.gov/files/drugs/published/In-Vitro-Metabolism–and-Transporter–Mediated-Drug-Drug-Interaction-Studies-Guidance-for-Industry.pdf, EMA DDI guidance 2012: https://www.ema.europa.eu/en/documents/scientific-guideline/guideline-investigation-drug-interactions-revision-1_en.pdf), then a clinical DDI study may be warranted.
2. *Indirect drug disease interactions*. Certain disease states have been identified where drug metabolizing enzyme or transporter expression and/or activity is significantly altered. For these diseases, when treatment results in a normalization of expression or activity levels back to the healthy range, there are implications for concomitant use of therapeutics with narrow therapeutic ranges. The second question to consider for siRNA DDI risk assessment is: Will the siRNA be used to alleviate a disease state that has significantly reduced drug metabolizing enzyme, transporter or siRNA-related protein activity? If the answer is ‘no’ then no further DDI assessment is warranted. If the answer is ‘yes’ since assessing potential downstream/upstream effects of siRNA in an *in vitro* assay may not be feasible, and since these effects may not be consistent across species (thus limiting the value of an *in vivo* preclinical DDI study), it is recommended that a clinical DDI study be considered.
3. *Direct mechanism-based interactions*. Givosiran is known to reduce the activity of CYP enzymes via its pharmacological activity. Thus, the third question to consider for the siRNA DDI risk assessment is: Are there any upstream or downstream effects of target gene knockdown that could potentially affect drug-metabolizing enzymes or transporters, or siRNA-related proteins such as ASGPR or Ago2? If the answer is ‘no’ then no further DDI assessment is warranted. However, if the answer is ‘yes’, since assessing potential downstream/upstream effects of siRNA in an *in vitro* assay may not be feasible, and since these effects may not be consistent across species (thus limiting the value of an *in vivo* preclinical DDI study), it is recommended that a clinical DDI study be considered.

Overall, the DDI potential for siRNAs is anticipated to be low. However, the chemical and/or pharmacological properties of some siRNAs may warrant *in vitro* and/or *in vivo* DDI assessments. A weight of evidence approach for including/excluding clinical DDI investigations, dependent on the properties of the siRNA, co-medications, and the pharmacology of the target, is recommended. Covariate analysis of co-medications using population PK modeling of clinical data may trigger dedicated DDI studies, however this has not yet been seen for currently approved siRNAs. In general, based on the regulatory filings to date, and in the absence of any pathway mediated DDI potential, it can be concluded that GalNAc–siRNA are unlikely to be a victim or perpetrator of DDIs relating to drug metabolizing enzymes or transporters , and *in vitro* or clinical investigations are not warranted.

## CONCLUSION

siRNA therapeutics have emerged as a novel class of medicines that are distinct from traditional small molecule drugs and biologics (Table 1). As such, thoughtful consideration is required to determine whether certain studies and reports, such as PPB evaluations and DDI risk assessments, add value to regulatory filing packages to help inform therapeutic index estimation, clinical development planning, and labeling. From a thorough review of data from publicly available regulatory filing documents and literature, along with experience from industry representatives, recommendations and decision trees intended to guide industry on Investigational New Drug and New Drug Application workflows were established. Specifically, recommendations are:

1. PPB evaluation is not advised when it does not aid in therapeutic index estimation, as in the case of GalNAc–siRNA. For other siRNA-containing therapeutic platforms including but not limited to, LNPs, peptide-siRNA conjugates, antibody-siRNA conjugates, lipid-siRNA conjugates, we recommend generating PPB data to set a regulatory precedence for a given class (*n* = 3 examples).
2. DDI risk assessment is not advised for GalNAc–siRNA except when the target RNA transcript or the protein it encodes is known or anticipated to play a role in the regulation or expression of a drug metabolizing enzyme, transporter, or siRNA-related protein. In addition, a risk assessment may be warranted when the disease state of the targeted patient population has an altered expression or activity profile of a drug metabolizing enzyme, transporter, or siRNA-related protein, and treatment of the disease is anticipated to normalize or otherwise modulate these profiles.

A major limitation of this work is that it is heavily weighted towards four approved siRNA from a single company. We conservatively recommend that, if in doubt, PPB evaluations and DDI risk assessments should be conducted for siRNA containing novel ligands, linkers and/or formulation excipients. In the future, once data accumulates and these moieties are no longer considered novel, as in the case of GalNAc–siRNA today, and if the aggregate data indicate that PPB and DDI studies would not aid in therapeutic index estimation or uncover DDI liabilities, respectively, then we similarly recommend that these studies not be performed.
